# 

**Published:** 1878-08-20

**Authors:** 


ORIGINAL AND SELECTED ARTICLES.

The Value of Extract of Malt and Loefland's Concentrated Liebig's Food for Infants, and an Account of their Introduction to the Medical Profession; by W. A. Greene, M. D., Macon, Ga....226
Laceration of the Penis; by A. A. Lyon, M.D., Shreveport, La....227
Management of Fevers in Germany; by W. S. Caldwell, M.D., Vienna, Austria....229
Typhoid Fever of Children; Ibid....280
The Therapeutical Use of Iodinized Milk....223
Case of Umbilical Hernia; by B. M. Walker, M.D., Danville, Va....234
Society Reports—Medico-Chirurgical Society.235

ABSTRACTS AND GLEANINGS.

Chloroform in Labor, 238; Enlarged Prostate, 239, Case Illustrating the Treatment of Bromide Rash with Arsenic, 240; Unofficinal Plants, 240; Medico-Legal Evidence, 242; Substitute for Mother's Milk, 243; Treatment of Acne Rosacea, 244; Hysteria in a Boy, 245; Theory of the Action of Quinine, 246; Lupus, 246; Intertrigo in Children, 246; Chloral in Dysentery, 247; A New Treatment of Tape-worm, 247; Morphological Changes in Syphilitic Blood, 247; Persistent Hiccough Treated Successfully by Pilocarpine, 247; The Use of Oleate of Mercury in Eye Diseases, 248; A Case of Hemorrhagic Coxitis Cured by Puncture, 248; Nitrate of Silver Injection in Chronic Dysentery, 248; Treatment of Cancer by Pressure, 248; Treatment of Remittent Fever, 249; Chloral and Glycerine in Constipation, 249; Thiersch o[illegible] Cauterization of Nævi, 249; Jaborandi in Obstinate Hiccough, 249.

PRACTICAL NOTES AND FORMULÆ.

Typho-Malarial Fever, 250, Rigid Os—Caution, 250; Salicylic Acid, 250; For Children, 250; To Prevent Diphtheria, 251; Nervous Palpitations, 251; Substitute for Mother's Milk, 251; For Colliquitive Diarrhœa, 251; Organic Heart Disease, 251; Cough Mixture, 251; Leucorrhœa, 251; Nervine and Antispasmodic, 252; Lactopeptine, 252; Tonic in Phthisis, 252; Castor Oil Emulsion, 252; Insects, 252; Summer Complaint, 252; Cinnamon in Hemorrhage, 252.

SCIENTIFIC ITEMS.

Frozen Air, 252; Dynamite, 252; Prevalence of Our Common Diseases in Japan, 253; Metals in the Sun, 253.

EDITORIAL AND MISCELLANEOUS.

Notice, 254; To Our Subscribers, 254; Is it Quackery, 253; Coca Erythroxylon, 255; Crawford W. Long, 255; Literary Notices, 255; Officers of the American Medical Association, 256; Ephraim McDowell, M.D., 256; Dermatological Association, 256; Death of Dr. Taylor, 256; A Nice Thing for a Doctor, 256; Our Advertising Department, 256.

